# Litter size influences rumen microbiota and fermentation efficiency, thus determining host early growth in goats

**DOI:** 10.3389/fmicb.2023.1098813

**Published:** 2023-01-20

**Authors:** Dangdang Wang, Guangfu Tang, Junjian Yu, Yuanyuan Li, Yannan Wang, Luyu Chen, Xinjian Lei, Yangchun Cao, Junhu Yao

**Affiliations:** College of Animal Science and Technology, Northwest A&F University, Yangling, China

**Keywords:** multiple litters, rumen microbiome, rumen fermentation, growth performance, goat

## Abstract

**Introduction:**

Multiple litters are accompanied by low birth weight, low survival rates, and growth rates in goats during early life. Regulating rumen microbiota structure can indirectly or directly affect host metabolism and animal growth. However, the relationship between high litter size and rumen microbiome, rumen fermentation, and growth performance in goat kids is unclear.

**Methods:**

In the present study, thirty 6-month-old, female goats were investigated, of which 10 goats were randomly chosen from single, twin and triplet goats respectively, and their birth weight was recorded. From birth, all goats were subjected to the same feed and management practices. Individual weaning and youth body weight were measured, and the rumen fluid samples were collected to characterize the bacterial communities and to determine the ruminal volatile fatty acids (VFA), free amino acids (AA), and free fatty acids (FA) concentration of those young goats.

**Results and Discussion:**

Compared with the single and twin goats, triplet goats have lower weaning and youth body weight and average daily gain (ADG). Ruminal propionate, butyrate, and total VFA were decreased in triplet goats. Meanwhile, ruminal AA, such as branched chain amino acids (BCAA), essential amino acids (EAA), unsaturated fatty acids (UFA), and monounsaturated fatty acids (MUFA) were decreased, while saturated fatty acids (SFA) and odd and branched chain fatty acids (OBCFA) were increased in triplet goats. Our results also revealed that litter size significantly affected the rumen bacterial communities, and triplet goats had a lower the Firmicutes: Bacteroidota ratio, the abundance of Firmicutes phylum, Rikenellaceae family, and *Rikenellaceae RC9 gut group*, and had a higher proportion of Prevotellaceae family, and several genera of Prevotellaceae, such as *Prevotella*, and *unclassified f Prevotellaceae*. Furthermore, Spearman’s correlation network analysis showed that the changes in the rumen bacteria were associated with changes in rumen metabolites. In conclusion, this study revealed that high litter size could bring disturbances to the microbial communities and decrease the rumen fermentation efficiency and growth performance, which can be utilized to better understand variation in microbial ecology that will improve growth performance in triplet goats.

## Introduction

The goat is one of the oldest domesticated animal species, and more than 1,000 breeds of goats around the world (MacHugh and Bradley, 2001; Lai et al., 2016). It provides a range of products and plays economically important roles in human productive activity (Haenlein, 2004; Pulina et al., 2018). In the goat industry, litter size is one of the most important economic traits (Akpa et al., 2011), and improvement of the reproductive traits are associated with large profits for farmers. However, the number of fetuses in the litter could affect the fetal growth rates and subsequent birth weight of goats (Gootwine et al., 2007; Zhang et al., 2008). Moreover, lambs born in multi-fetus litters have lower pre- and postnatal survival rates of the goats (Christley et al., 2003). Therefore, the detrimental impact of multiple litters on the birth weight and growth performance of goat kids should be more thoroughly studied.

Trillions of microbes colonize in the rumen and play crucial roles on metabolism and ruminant growth. This microbial cohort contains cellulolytic, hemicellulolytic, amylolytic, proteolytic, and lipolytic species, which ferment feedstuff to yield volatile fatty acids (VFA), free fatty acids (FA), and bacteria protein (O’Hara et al., 2020). Those products of rumen microbial fermentation can meet most of the host’s energy and nutrition needs (Matthews et al., 2019). Recent studies found that the presence of a microbiome in the gut of fetal lambs (Bi et al., 2021), and maternal gut and reproductive tract microbiome may affect the fetal microbiome and health (Rowe et al., 2020; Hummel et al., 2021). Furthermore, studies also found that modulating the pregnant sow’s microbiome could improve reproductive efficiency (Jiang et al., 2019) and piglet survival rate (Ma et al., 2020). However, little is known regarding the impact of litter size on early life programming of rumen microbiome and its implication for goat growth.

In this study, a total of 30 newborn female kids were selected from 30 litters, one female kid per litter. Ten were randomly selected from single (litter size = 1[LZ1]), twins (litter size = 2[LZ2]), and triplets (litter size = 3[LZ3]) for sampling, respectively. The aim of this study was to (1) evaluate the effects of litter size on weaning and youth growth performance of goat kids, (2) investigate the impact of litter size on rumen fermentation [VFA, amino acids (AA), and FA] and rumen microbial communities of young goats, and (3) reveal the relationship between the litter size related rumen microbiota and microbial metabolites, and growth performance traits of young goats.

## Materials and methods

### Ethics statement

In the present study, all animal procedures were approved by the Institutional Animal Care and Use Committee of Northwest A&F University.

### Animals and sample collection

Field experiment were performed at a Saanen goat farm in Baoji, Shaanxi (34°41′N, 109°09′E). Thirty ewes delivering in 1 week (third birth), including 10 ewes producing single, 10 ewes producing twins, and 10 ewes producing triplets, with each litter including at least one female newborn, were selected. After born, one female kid was chosen from each litter, 30 female kids in total, were randomly selected from these 30 litters for sampling. Briefly, 10 female kids were randomly selected from single, twins, and triplets, respectively, and named as LZ1, LZ2, and LZ3. And the birth weight of those kids was weighed within 12 h after birth. And then, all kids were transferred to the kid barn with bottle-feeding of mixed milk collected from ewes. During pre-weaning phase (~90 days old), all goats were feed milk, alfalfa hay, and concentration mixture. After weaning, the goats were fed TMR with a 60:40 forage to concentration ratio. The animals were fed three times daily at 0730, 1300, and 1900 h. Water was available *ad libitum*. The detailed feeding programs and ingredient compositions are shown in Supplementary Table S1.

The body weight, wither height, body length, and heart girth of weaning and 6-month-old (188.9 ± 0.4 days old, mean ± SE) of those three group goats were measured. And the rumen fluid sample of those young goats were collected *via* esophageal tube before morning feeding. Briefly, the first ~50 ml of rumen fluid was discarded to avoid saliva contamination, and the text 30 ml rumen fluid strained through four layers of sterile cheesecloth under a constant flux of CO_2_. Then the rumen fluid was aliquoted and stored at −80°C for further analysis.

### Dry matter intake measurements

The feed intake of all goats was measured 1 week before sampling (171.9 ± 0.4 days old). In brief, feed offered to and refused by each goat was recorded continuously for 7 days. The feed samples were dried at 65°C for 48 h to obtain dry matter content of ration. Daily dry matter intake (DMI) per goat was calculated by multiplying daily as-fed intake by dry matter content of ration.

### Ruminal VFA assay

Ruminal VFA (acetate, propionate, butyrate, valerate, isobutyrate, and isovalerate) analysis were performed through separation and quantification by a GC (Agilent 7820A, Santa Clara, CA, United States) with a capillary column (AE-FFAP of 30 m × 0.25 mm × 0.33 μm; ATECH Technologies Co., Lanzhou, China; Li et al., 2014). Briefly, thawed rumen fluid samples were centrifuged for 10 min at 13,500 rpm at 4°C. To remove the protein, a 1.5 ml supernatant was mixed with 300 μl 25%w/v metaphosphoric solution. After standing for 4 h at 4°C, the mixture was centrifuged for 10 min at 13,500 rpm at 4°C. Then 1 ml supernatant was moved into 200 μl 25% crotonic acid, and the mixture was collected into an EP tube passed through a 0.45-μm filter.

### Ruminal free AA assay

The detection of ruminal free amino acids abundance were conducted using LC–MS/MS (Exion LC AC, QTRAP 5500, AB SCIEX, Framingham, MA, United States) according to method described as previously described (Chen et al., 2022). In brief, in order to precipitate proteins rumen fluid samples were mixed through with sulfosalicylic acid (10%, wt/vol) and centrifuged at 3,000 × *g* for 10 min at 4°C. Then the supernatant fluid was collected and filtered through a 0.45-μm filter.

### Ruminal FA assay

Ruminal fatty acids composition was analyzed using the method of Sun and Gibbs (2012). The freeze-dried sample (0.3–0.4 g) was methylated with 4 ml of 0.5 mol/L NaOH/methanol for 15 min at 50°C. Then the mixture was added 4 ml 2% HCl/methanol (v/v) and vortexed for 5 min, followed by a water bath (50°C) for 60 min. The extract was dissolved in 2 ml of heptane and then introduced to the GC (Agilent Technologies 7820A GC system, Santa Clara, CA, United States) equipped with a fused silica capillary column (SP-2560, 100 m × 0.25 mm × 0.2 μm; Supelco Inc., Bellefonte, PA, United States).

### Bacterial DNA extraction and PCR amplification

The total DNA was extracted using the QIAamp DNA Stool Mini kit (QIAGEN, Germany) according to the manufacturer’s protocol. The DNA concentration was determined by using a Nanodrop-2000 (Thermo Fisher Scientific, United States), and the purity was monitored on 1% agarose gel electrophoresis. The DNA was stored at −80°C until further processing. The amplicon library preparation was performed by polymerase chain reaction amplification of the V3-V4 region of the 16S rRNA gene using the primer pairs: 338F (5′-ACTCCTACGGGAGGCAGCAG-3′) and 806R (5′-GGACTACHVGGGTWTCTAAT-3′). The PCR program was carried as previously described (Xue et al., 2018). Paired-end (2 × 300 bp) sequencing was performed on Illumina platform (Illumina, United States) according to the standard protocols (Caporaso et al., 2012).

### 16S rRNA sequencing analysis

The raw sequences were merged with FLASH (v1.2.11; Magoč and Salzberg, 2011) and quality filtered with fastp (0.19.6; Chen et al., 2018). Sequences were demultiplexing using QIIME2 v2021.8, and the construction of an amplicon sequence variant (ASV) table using DADA2 (Callahan et al., 2016). Bacterial 16S ASVs were assigned a taxonomy using the SILVA database v138 as the reference.

The alpha diversity diversities of the bacterial communities were determined using various diversity indices (Sobs, ACE, Chao 1, Shannon, Simpson) and calculated using the procedures within QIIME 2. The NMDS (non-metric multidimensional scaling) graphs were performed based on Bray-Curtis distance and statistical significance was determined using analysis of similarities (ANOSIM) with 999 permutations at the ASV level.

### Statistical analysis

Differential DMI, growth performance traits (body weight, ADG, wither height, body length, and heart girth), and ruminal metabolites (VFA, AA, and FA) were tested by one-way ANOVA among three groups, and Student’s *t*-test was used between two groups. Orthogonal polynomials were used to determine the linear effects of increasing the litter size.

The taxa with different relative abundances among those three groups were identified by a Kruskal–Wallis test, and Wilcoxon rank-sum test was used between two groups with a false discovery rate (FDR) value <0.05 to correct the *p* values. The differences were statistically significant at *p* < 0.05 or a tendency of difference at *p* < 0.10. Spearman’s rank correlation coefficients were used to examine the correlations between bacterial abundance and rumen metabolites concentrations.

## Results

### Growth performance

In the present study, although there was no significant difference in birth weight between these three groups (*p* > 0.05). Numerically, the LZ3 group (2.94 ± 0.20 kg) with lower birth weight compared to LZ1 (3.21 ± 0.14 kg) and LZ2 (3.20 ± 0.12 kg) group (Supplementary Figure S1A).

**Figure 1:**
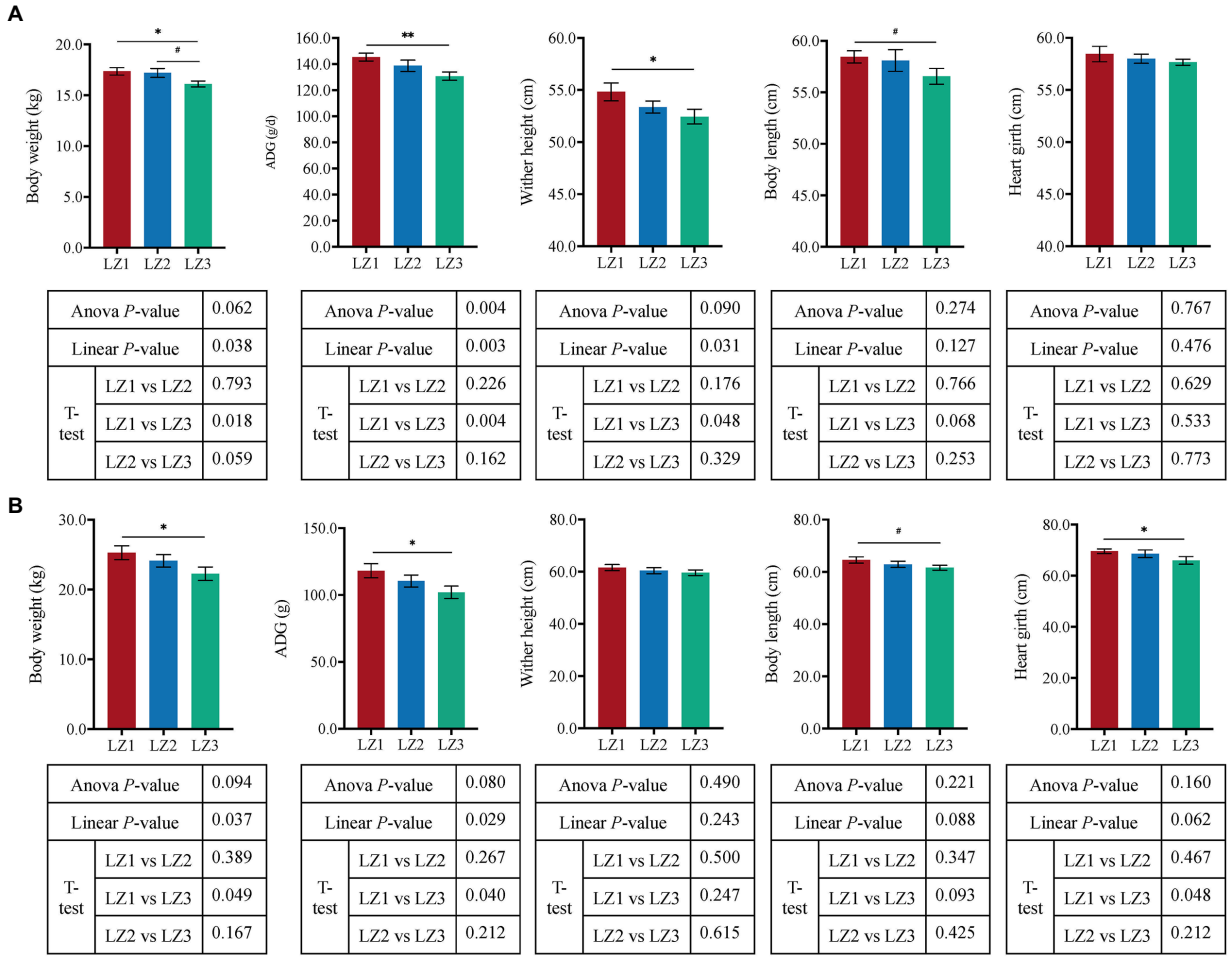
Effect of litter size on growth performance of weaning **(A)** and young **(B)** goats. Data are presented as mean± SEM, ^#^*p* < 0.1, *p < 0.05, **p < 0.01.

The growth performance traits were shown in Figure 1. With the increased litter size, the weaning weight, ADG, and wither height of goat kids were linearly decreased (*p* < 0.05). Moreover, compared to the LZ1 group, the weaning weight, ADG, and wither height were significantly lower in LZ3 group (*t*-test, *p* < 0.05).

And, there was no significant difference in DMI between the three groups (*p* = 0.729, Supplementary Figure S1B) at the youth period. With the increased litter size, the youth body weight, and ADG of young goats were linearly decreased (*p* < 0.05), the body length and heart girth had a tendency to decrease (*p* < 0.1). Furthermore, compare to the LZ1 group, LZ3 group had a lower body weight, ADG, heart girth (*t*-test, *p* < 0.05), and body length (*t*-test, *p* = 0.093) at the youth period.

### Ruminal fermentation parameters

As shown in Table 1, with the increased litter size, the concentration of ruminal propionate, total VFA of young goats were linearly decreased (*p* < 0.05), the ruminal butyrate concertation had a tendency to decrease (*p* = 0.098). In addition, compare to the LZ1 group, LZ3 group had a lower propionate, total VFA (*t*-test, *p* < 0.05), and butyrate concentration (*t*-test, *p* = 0.093).

**Table 1:** Effect of litter size on ruminal VFA (mM) concentration of young goats.

Items	Groups			SEM	ANOVA	Linear	*T*-test		
	LZ1	LZ2	LZ3				LZ1 vs. LZ2	LZ1 vs. LZ3	LZ2 vs. LZ3
Acetate	65.41	61.94	60.81	1.711	0.533	0.296	0.453	0.136	0.821
Propionate	23.32	18.44	15.85	1.244	0.041	0.015	0.084	0.030	0.679
Isobutyrate	0.83	0.82	0.74	0.040	0.571	0.317	0.839	0.376	0.378
Butyrate	10.89	9.99	8.41	0.587	0.255	0.098	0.546	0.093	0.279
Isovalerate	1.48	1.56	1.35	0.074	0.566	0.562	0.652	0.472	0.346
Valerate	0.95	0.86	0.84	0.038	0.487	0.268	0.335	0.252	0.872
Total VFA	102.89	93.59	88.01	2.981	0.058	0.019	0.140	0.021	0.343

Different letters (a, b) within a row means values with significantly different (*p* < 0.05). LZ1, single goat; LZ2, twin goats; LZ3, triplet goats; SEM, standard error of mean; VFA, volatile fatty acids.

Then the difference in ruminal AA and FA were identified between the three groups. We found that the concentration of some essential amino acids (EAA), such as His, Trp, Lys, Phe, Val and total EAA concentration, were linearly decreased with the increased litter size (Table 2, *p* < 0.05). In addition, compared to the LZ1 group, the concentration of His, Cys, Val, Trp, Phe, Lys, and total EAA were significantly decreased in LZ3 group (*t*-student, *p* < 0.05), and the concentration of some AA, such as Leu, Pro, and total branched chain amino acids (BCAA) of LZ3 group, tended to be decreased than LZ1 group (*t*-student, *p* < 0.1).

**Table 2:** Effect of litter size on ruminal free AAs (μmol/L) concentration of young goats.

Items	Groups			SEM	ANOVA	Linear	*T*-test		
	LZ1	LZ2	LZ3				LZ1 vs. LZ2	LZ1 vs. LZ3	LZ2 vs. LZ3
Asp	676.37	648.45	477.92	50.957	0.383	0.192	0.844	0.178	0.196
Glu	493.67	386.59	308.05	43.990	0.365	0.168	0.432	0.197	0.400
Ser	510.03	350.57	272.34	41.714	0.111	0.042	0.172	0.052	0.347
His	218.90	151.60	88.51	20.520	0.079	0.026	0.248	0.015	0.131
Gly	623.72	435.52	314.51	54.070	0.121	0.044	0.221	0.05	0.221
Thr	312.16	238.87	180.60	31.639	0.165	0.064	0.192	0.09	0.499
Arg	55.66	73.68	69.06	7.211	0.629	0.482	0.335	0.539	0.860
Ala	1241.27	842.05	689.11	103.339	0.139	0.056	0.182	0.063	0.412
Tyr	325.64	247.81	184.84	25.144	0.142	0.051	0.271	0.059	0.203
Cys	103.17	71.43	50.32	8.397	0.074	0.025	0.169	0.022	0.236
Val	498.42	400.84	304.39	43.533	0.119	0.043	0.216	0.042	0.253
Met	228.32	199.71	147.64	22.096	0.253	0.102	0.399	0.106	0.264
Trp	52.81^a^	54.77^a^	25.69^b^	4.886	0.039	0.022	0.765	0.007	0.017
Phe	223.70	193.13	142.13	18.803	0.140	0.050	0.296	0.045	0.189
Ile	409.59	371.27	279.01	38.193	0.284	0.117	0.443	0.116	0.270
Leu	520.57	442.25	349.68	43.029	0.168	0.064	0.269	0.058	0.287
Lys	1146.40	907.48	691.25	100.219	0.109	0.039	0.199	0.043	0.236
Pro	182.17	124.67	116.13	11.297	0.070	0.036	0.054	0.058	0.783
BCAA	1541.47	1214.36	933.08	115.570	0.184	0.069	0.318	0.056	0.268
EAA	3971.65	3033.60	2277.97	294.093	0.125	0.044	0.254	0.040	0.226
NEAA	4155.95	3107.08	2413.21	314.136	0.140	0.052	0.236	0.055	0.277

Different letters (a, b) within a row means values with significantly different (*p* < 0.05). LZ1, single goat; LZ2, twin goats; LZ3, triplet goats; SEM, standard error of mean; Asp., aspartic acid; Glu, glutamic acid; Ser, serine; His, histidine; Gly, glycine; Thr, threonine; Cys, cysteine; Val, valine; Met, methionine; Trp, tryptophan; Phe, phenylalanine; Ile, Isoleucine; Leu, leucine; Lys, lysine; Pro, proline; BCAA, branched chain amino acids; EAA, essential amino acids; NEAA, non-essential amino acids.

As shown in Table 3, the proportion of iso C14:0, anteiso C15:0, C15:0, saturated fatty acids (SFA), odd and branched chain fatty acids (OBCFA) and the ratio of SFA to UFA were linearly increased with the increased litter size (*p* < 0.05). And the proportion of C16:0, C18:1, C18:1t11, C18:1c9, unsaturated fatty acids (UFA) and monounsaturated fatty acids (MUFA) were linearly decreased with the increased litter size (*p* < 0.05). In addition, we found that the proportion of iso C14:0, anteiso C15:0, C15:0, OBCFA were significantly increased and C16:0, C18:1t11, C18:1c9, C18:1, and MUFA were significantly decreased in the LZ3 group compared to the LZ1 group.

**Table 3:** Effect of litter size on ruminal FAs (g/100 g of total FAs, relative abundance >0.1%) of young goats.

Items	Groups			SEM	ANOVA	Linear	*T*-test		
	LZ1	LZ2	LZ3				LZ1 vs. LZ2	LZ1 vs. LZ3	LZ2 vs. LZ3
C12:0	1.19	1.38	1.66	0.133	0.438	0.207	0.491	0.193	0.501
iso-C14:0	0.75	1.28	1.32	0.110	0.060	0.036	0.046	0.008	0.898
C14:0	3.72	3.70	4.63	0.339	0.523	0.356	0.976	0.343	0.369
iso-C15:0	2.07	2.84	2.64	0.166	0.121	0.132	0.053	0.109	0.676
anteiso-C15:0	4.38^b^	6.12^a^	5.87^a^	0.270	0.013	0.024	0.003	0.018	0.739
C15:0	1.96	2.47	2.60	0.126	0.106	0.048	0.073	0.033	0.719
C16:0iso	2.14	2.48	2.73	0.178	0.456	0.217	0.401	0.225	0.62
C16:0	43.66	40.35	40.55	0.704	0.083	0.061	0.061	0.037	0.913
anteiso-C17:0	2.46	2.46	2.31	0.075	0.741	0.506	0.998	0.201	0.556
C18:0	5.45	7.57	7.05	0.499	0.204	0.194	0.094	0.081	0.741
C18:1t11	3.84^a^	2.96^b^	2.26^b^	0.175	0.001	<0.001	0.015	0.002	0.074
C18:1c9	14.57^a^	11.29^b^	10.97^b^	0.516	0.005	0.003	0.002	0.009	0.789
C18:1c11	2.88	1.59	2.99	0.289	0.084	0.912	0.014	0.902	0.089
C18:2cis9,12	5.62	5.04	5.34	0.367	0.799	0.728	0.501	0.771	0.768
C18:1	21.26^a^	16.90^b^	17.49^b^	0.604	0.002	0.003	<0.001	0.015	0.646
SFA	71.25^b^	75.60^a^	74.71^a^	0.676	0.010	0.018	0.003	0.052	0.558
UFA	28.75^a^	24.40^b^	25.29^b^	0.676	0.010	0.018	0.003	0.052	0.558
SFA/UFA	2.52^b^	3.05^a^	3.03^a^	0.096	0.027	0.020	0.005	0.054	0.949
SCFA	0.35	0.78	0.51	0.085	0.089	0.295	0.046	0.172	0.33
MCFA	62.22	64.26	63.94	0.773	0.517	0.364	0.253	0.356	0.897
LCFA	37.43	34.96	35.27	0.762	0.354	0.244	0.139	0.268	0.895
MUFA	22.80^a^	17.97^b^	18.60^b^	0.601	<0.001	0.001	<0.001	0.005	0.623
PUFA	6.50	6.43	6.68	0.300	0.947	0.846	0.915	0.818	0.756
OBCFA	15.33	18.98	19.44	0.833	0.082	0.043	0.053	0.034	0.842

Different letters (a, b) within a row means values with significantly different (*p* < 0.05). LZ1, single goat; LZ2, twin goats; LZ3, triplet goats; SEM, standard error of mean; SFA, saturated fatty acids; UFA, unsaturated fatty acids; SCFA, short chain fatty acids; MCFA, medium chain fatty acids; LCFA, long chain fatty acids; MUFA, monounsaturated fatty acids; PUFA, polyunsaturated fatty acids; OBCFA, odd and branched chain fatty acids.

Together, our results revealed that with the increased litter size, the growth performance and rumen fermentation efficiency were linearly decreased.

### Ruminal microbial diversity and structure

Based on the above results, the difference in rumen microbiota between LZ3 group and LZ1, LZ2 group were identified, to evaluate whether alterations of ruminal fermentative capacity were influence by rumen microbiota.

After size filtering, quality control, and chimera removal using the QIIME 2 pipeline, an average of 45,662 reads were generated for bacteria from a total 1,369,847 quality reads from 30 rumen fluid samples, with an average length of 416 bp. The calculated Good’s coverage exceeded 99.9% for all samples.

**Figure 2:**
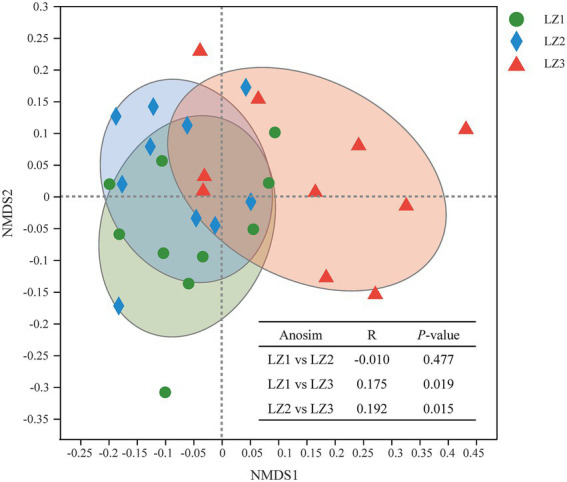
Non-metric multidimensional scaling plot based on ASV level to identify differences in microbial community structure among different groups.

To compare bacterial community diversity across different groups, alpha-diversity and beta-diversity were evaluated. There were no significant differences in Sobs index, Ace index, Chao 1 index, Shannon index, and Simpson index among the three groups (Table 4, *p* > 0.05). And there were no significant difference in alpha-diversity indices between LZ1 and LZ3 group (*p* > 0.05). In a beta-diversity analysis (NMDS based on Bray-Curtis), the LZ1 and LZ2 group were clustered together and could not be distinguished (*p* = 0.477), whereas the LZ3 group was clearly distinguished from the LZ1 and LZ2 group (Figure 2, *p* < 0.05).

**Table 4:** Alpha-diversity comparisons in the rumen microbiota among the different groups.

Index	Groups			SEM	Kruskal–Wallis test	Wilcoxon rank sum test		
	LZ1	LZ2	LZ3			LZ1 vs. LZ2	LZ1 vs. LZ3	LZ2 vs. LZ3
Sobs	566.5	611.5	645.6	21.92	0.309	0.341	0.119	0.676
Ace	571.2	616	650.7	22.21	0.309	0.341	0.119	0.676
Chao1	571.5	615.2	650.1	22.26	0.309	0.341	0.119	0.676
Shannon	5.11	5.3	5.31	0.080	0.487	0.309	0.305	0.909
Simpson	0.025	0.016	0.02	0.003	0.321	0.108	0.790	0.425

LZ1, single goat; LZ2, twin goats; LZ3, triplet goats; SEM, standard error of mean.

### Ruminal microbial composition

In total, 21 phyla, 32 classes, 71 orders, 120 families, and 237 genera were identified. More specifically, Firmicutes (50.2%), Bacteraioidota (43.1%), and Patescibacteria (3.4%) were the predominant phyla. The abundance of Firmicutes in LZ3 group was significantly lower than LZ2 group, while the abundance of Bacteraioidota in LZ3 group was significantly higher compared to LZ1 and LZ2 group (*p* < 0.05). Meanwhile, the Firmicutes: Bacteraioidota ratio was significantly decreased in the LZ3 group (Supplementary Figure S2, *p* < 0.05).

**Figure 3:**
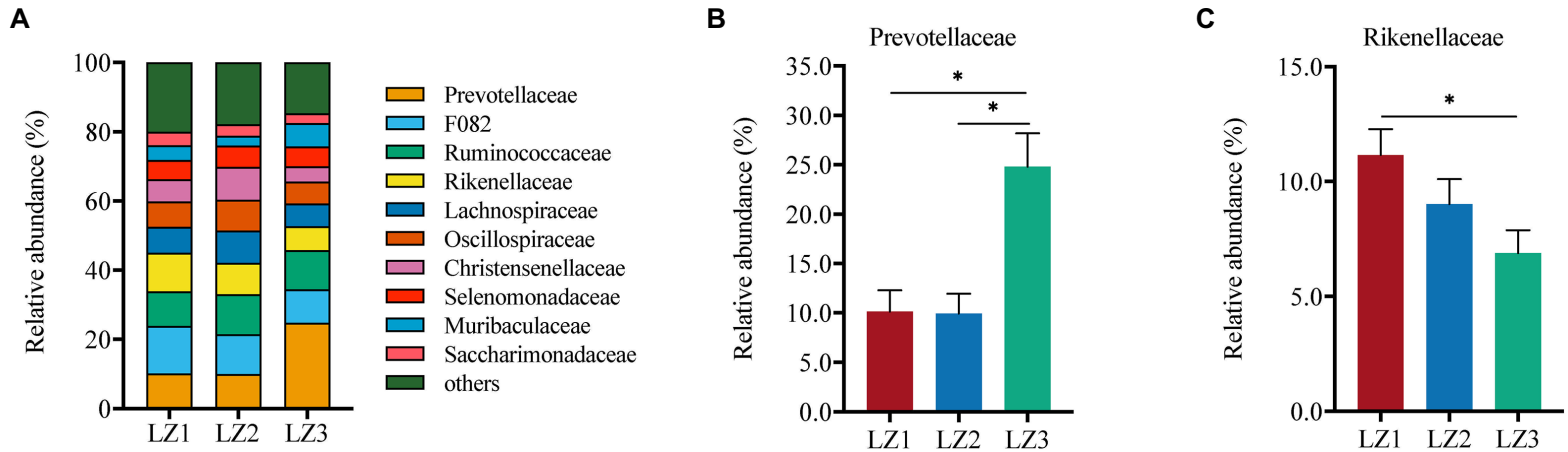
Compositions of the rumen microbiota among the different groups at family level. **(A)** Relative abundance of major family, **(B)** Prevotellaceae, **(C)** Rikenellaceae. Data are presented as mean ± SEM, *p < 0.05, **p < 0.01.

**Figure 4:**
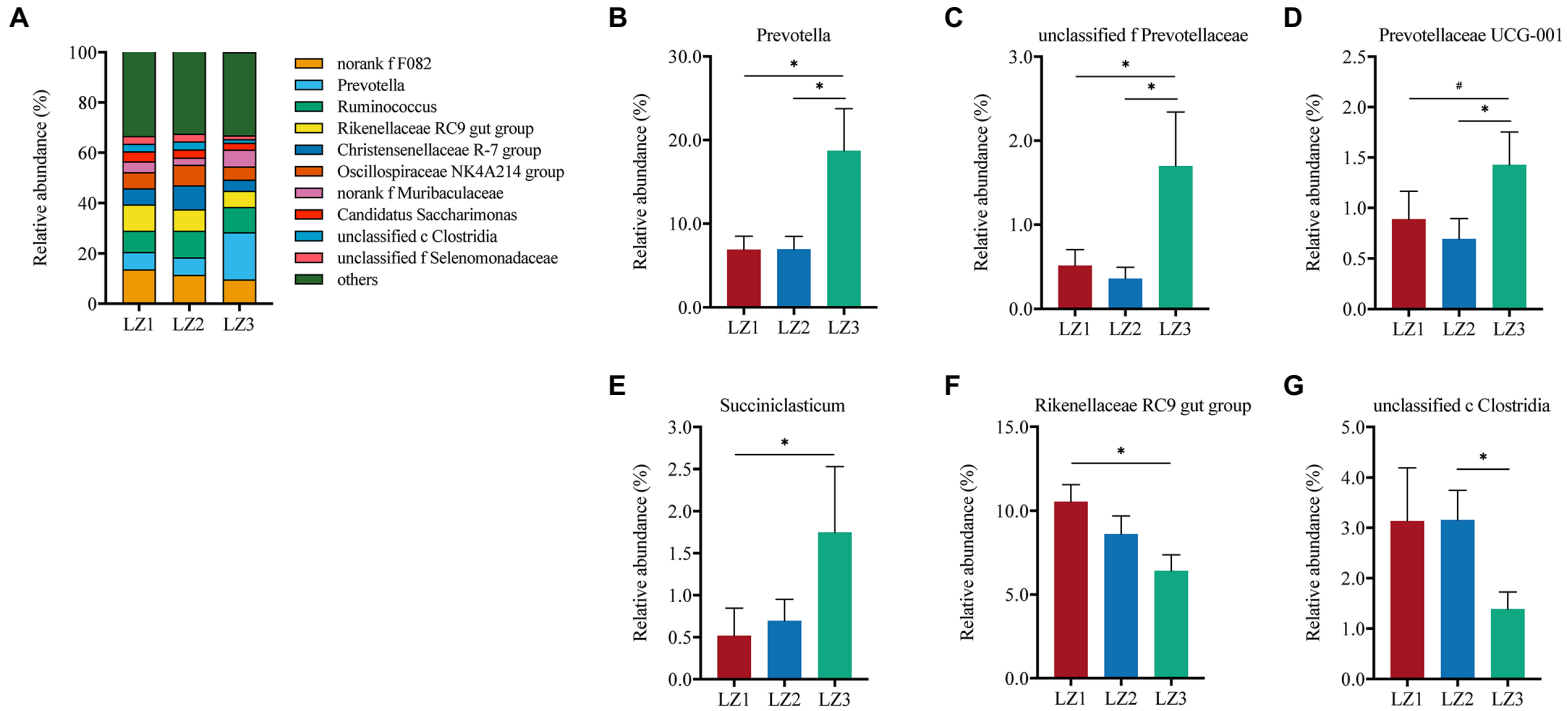
Composition of the rumen microbiota among the different groups at genus level. **(A)** Relative abundance of major genus, **(B)** *Prevotella*, **(C)** *unclassified f Prevotellaceae*, **(D)** *Prevotellaceae UCG-001*, **(E)** *Succiniclasticum*, **(F)** *Rikenellaceae RC9 gut group*, **(G)** *unclassified c Clostridia*. Data are presented as mean ± SEM, ^#^*p* < 0.1, *p < 0.05.

As shown in Figure 3, Prevotellaceae (24.8%) was the most abundant family in LZ3 group. While, F082 was the most abundant family in LZ1 (13.7%) and LZ2 (11.5%) group. Prevotellaceae abundance in LZ3 group was significantly higher than that in LZ1 and LZ2 group (*p* < 0.05). Conversely, the relative abundance of Rikenellaceae in LZ3 group was significantly lower than that in LZ1 group (*p* < 0.05). At the genus level, *norank f F082* (11.6%), *Prevotella* (10.9%), *Ruminococcus* (9.6%), *Rikenellaceae RC9 gut group* (8.5%), *Christensenellaceae R-7 group* (6.8%), *Oscillospiraceae NK4A214 group* (6.6%), norank f *Muribaculaceae* (4.6%), *Candidatus Saccharimonas* (3.4%), *unclassified c Clostridia* (2.6%), and *unclassified f Selenomonadaceae* (2.5%) were the dominant (Figure 4). The abundance of *Prevotella*, *unclassified f Prevotella* and *Prevotellaceae UCG-001* in LZ3 group were significantly higher than that in LZ1 and LZ2 group (*p* < 0.05). *Succiiniclasticum* abundance in the LZ3 group were significantly higher than that in LZ1 group (*p* < 0.05). Conversely, the abundance of *Rikenellaceae RC9 group* and *unclassified c Clostridia* were significantly lower than that in LZ1 or LZ2 group (*p* < 0.05).

### The correlation between ruminal metabolites and ruminal microbiota.

**Figure 5:**
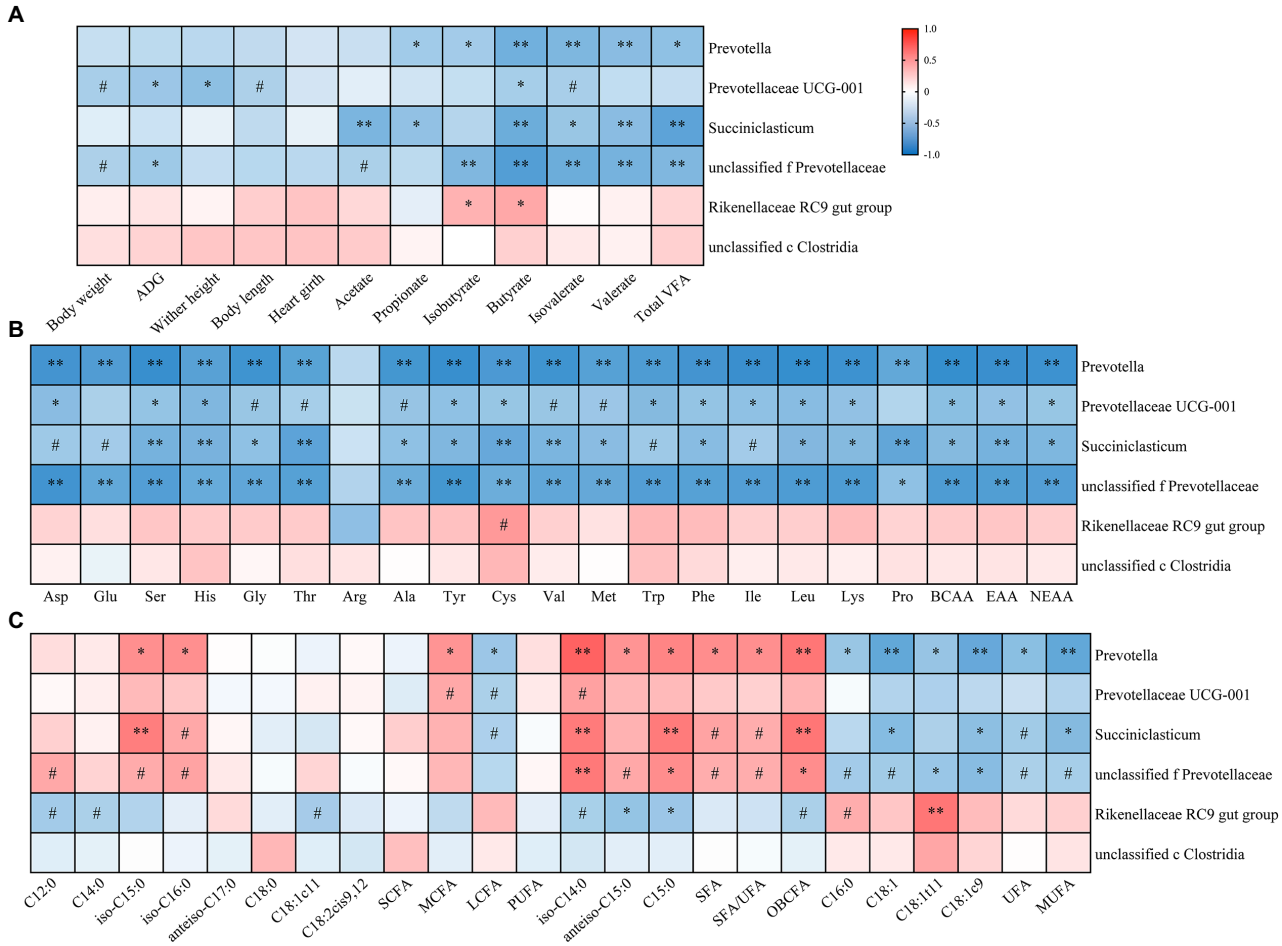
Heatmap showing association between growth performance, ruminal metabolites and rumen microbiota of young goats. The relationship between rumen microbiota and growth performance, rumen VFA **(A)**, AA **(B)**, and FA **(C)** of young goats. The color gradient represents the values of correlation coefficients. Red and blue indicate positive and negative correlations, respectively. ^#^*p* < 0.1, *p < 0.05, **p < 0.01.

As shown in Figure 5A, those LZ3 enriched bacteria were negatively correlated with ruminal VFA concentration and growth performance traits of young goats. For example, the relative abundance of *Prevotellaceae UCG-001* was negatively correlated with ADG, wither height, and ruminal butyrate concentration (*p* < 0.05). The relative abundance of *Prevotella*, and *Succinniclasticum* were negatively correlated with ruminal propionate, butyrate, valerate, and total VFA concentration (*p* < 0.05). And *Rikenellaceae RC9 gut group* had a significantly positively correlated with ruminal isobutyrate and butyrate concentration (*p* < 0.05).

And then, we analyzed the correlation between ruminal AA, FA, and litter size related bacteria genera (Figures 5B,C). The results showed that those LZ3 enriched bacteria were significantly negatively correlated with ruminal AA, UFA, and MUFA, such as *Prevotella* and ruminal BCAA, total EAA, anteiso C15:0, and OBCFA were negatively correlated but positively correlated with C18:1t11, UFA, and MUFA (*p* < 0.05). *unclassified f Prevotellaceae* were significantly negatively associated with almost all ruminal AA, such as Leu, His and total BCAA, and C18:1t11 but positively associated with OBCFA (*p* < 0.05).

## Discussion

Litter size is a critical and complicated economic trait within the goat industry. Small ruminants have a great frequency of multiple births, and past research has shown that the average litter size in goats varies from 1.30 to 2.37 (de Lima et al., 2020). However, few studies focus on the effect of high fertility on rumen fermentation and growth performance of offspring. In the present study, we investigated the relationship between litter size and rumen fermentation, microbiota community, and growth performance of young goats. We found that LZ3 goats (triplets) have lower growth performance and rumen fermentation efficiency. Moreover, rumen microbiota structure and composition between LZ3 group and LZ1, LZ2 group were different. And these differences in rumen microbiome were associated with a decrease in rumen metabolites, such as propionate, total VFA, and EAA.

Rumen microbiota plays a fundamentally important role in the development of rumen function and metabolism at the host. Previous studies confirmed the correlations of ruminal microbial features with the ruminants’ phenotypic characteristics, such as rumen fermentation products (Kamke et al., 2016; Wallace et al., 2019), feed efficiency (Myer et al., 2015; Shabat et al., 2016; Xue M.-Y. et al., 2022), and milk production (Jami et al., 2014; Xue et al., 2020). According to our current study, triplet kids lead to significant alterations in the microbiota structure of rumen, which had lower pre-weaning and youth growth performance.

Similar to previous studies (Jami et al., 2013; Li et al., 2019; Tong et al., 2022), we also observed that Firmicutes, and Bacteraioidota were the predominant bacterial phylum in the ruminal ecosystem. The abundance of Firmicutes, Bacteraioidota, and the ratio of Firmicutes to Bacteraioidota were different in different litter sizes. The phyla Firmicutes and Bacteraioidota were known for polysaccharide fermentation. And Wang et al. (2019) found that an improved intestinal Firmicutes: Bacteraioidota ratio could allow the host to absorb more energy from the diet and to store energy.

Moreover, we found that rumen fermentation products, such as total VFA, propionate, butyrate, EAA, and BCAA concentrations, which were decreased in triplet goats, had negatively associated with those members of Prevotellaceae family. Rumen VFA and EAA are the major energy and nutrients for animal growth, health and production (Maeng et al., 1976; Van Houtert, 1993; Xue et al., 2019). His and Trp were the limiting AA for growing goats (Onodera, 2003; Ma et al., 2010), and some cell experiments demonstrated that the addition of His activated the mTOR pathway, which up-regulated phosphorylation of the downstream protein and ultimately prompted protein synthesis (Appuhamy et al., 2012). In the current study, the abundance of Prevotellaceae family and several genera of it, such as *Prevotella*, *unclassified f Prevotellaceae*, *Prevotellaceae* UCG-001, were higher in triplet goats. *Prevotella* species had a documented role in metabolism of carbohydrates, such as hemicellulose, starch, xylan, and pectin (Kabel et al., 2011; De Filippis et al., 2019), and nitrogen (Kim et al., 2017). Recent studies suggested the abundance of *Prevotellaceae* family was negatively correlated with feed efficiency in ruminants (Paz et al., 2018; Zhang et al., 2021). Rumen *Prevotella,* and *Prevotellaceae UCG-001* abundance were reported to be negatively correlated with propionate and AA concentrations (Bi et al., 2018; Tong et al., 2020; Liu et al., 2022; Xue B. et al., 2022), and higher in inefficient animals (Carberry et al., 2012; Mccann et al., 2014).

Dietary lipid plays an important role in rumen metabolism and animal production. Moreover, dietary FA are extensively hydrogenated and isomerized by rumen microbes. These transformations directly determine milk and meat FA composition, which is a criterion of the nutritional quality of the products (Chilliard et al., 2007). We observed that ruminal OBCFA and its main constituent FA, anteiso C15:0, were higher in triplet goats. OBCFA are synthesized by ruminal bacteria using the precursors, such as propionate, valerate, BCAA, and incorporated in their cell membrane (Vlaeminck et al., 2006). A previous study showed that rumen cellulolytic bacteria contain relatively high amounts of OBCFA (Fievez et al., 2012). This is consistent with our finding that those members of *Prevotellaceae* family (mainly cellulolytic bacteria), which were enriched in the rumen of triplet goats, were positively associated with OBCFA. Therefore, these triplets enriched bacteria may produce less ruminal VFA, and AA, and utilize more propionate, and BCAA to produce OBCFA, which leads to less energy and nutrient supply for animal growth in triplet goats.

Ruminal butyrate concentration, the proportion of C16:0, C18:1 components (such as C18:1, C18:1t11, and C18:1c9), and MUFA were lower in triplet goats. UFA, especially MUFA, have taxic effects on rumen methanogenic archaea, which lead to the reduction of methane production (Beauchemin et al., 2008). An *in vitro* study found that oleic acid (C18:1c9) supplementation in diet can reduce methane production (Wu et al., 2016). Moreover, the results showed that the abundance of *Rikenellaceae RC9 gut group* was lower in the rumen of triplet goats, which were positively correlated with ruminal butyrate, C16:0, C18:1t11, and negatively correlated with ruminal anteiso C15:0 and OBCFA. *Rikenellaceae RC9 gut group* can reportedly degrade structural carbohydrates and starch in the rumen (Asma et al., 2013), and be involved in the production of VFA (Graf, 2014) and the scavenging of H_2_. A recent study found that the *Rikenellaceae RC9 gut group* abundance was associated with an increase of the feed efficiency trait (Andrade et al., 2022). These bacteria may play roles in rumen fermentation and animal growth. Based on the results, it would be promising to develop probiotics that promote the “single-enriched” and inhibit the “triplets-enriched” bacterial taxa to improve rumen efficiency and growth rates in young goats.

## Conclusion

In conclusion, the present study disclosed the differences in rumen microbiota composition, rumen fermentation, and growth performance between single, twin, and triplet goats. Our study revealed that triplet goats have lower growth performance and rumen fermentation efficiency. The Prevotellaceae family, and several genera of Prevotellaceae, such as *Prevotella*, were higher in the rumen of triplet goats, whereas the abundance of *Rikenellaceae RC9 gut group* was lower in the rumen of triplet goats. Our results revealed the change in rumen microbiome composition may affect the rumen metabolism, thus slowing the growth rates in triplet goats. Overall, our study generated relevant information for manipulating rumen microbiome and improving rumen function that will increase the feed efficiency and growth performance in goats.

## Data availability statement

The datasets presented in this study can be found in online repositories. The names of repository/repositories and accession number can be found in the NCBI sequence read archive, accession number PRJNA918955.

## Ethics statement

The animal study was reviewed and approved by Institutional Animal Care and Use Committee of Northwest A&F University.

## Author contributions

DW and JYa designed the research. DW, JYu, YL, and YW performed the experiment. DW, GT, and LC analyzed the data. DW wrote the manuscript. YC and XL undertook revision work. JYa finalized the manuscript. All authors read and approved the final version of the manuscript.

## Funding

The present study was supported by the National Natural Science Foundation of China (32072761 and 32272829), Chinese Universities Scientific Fund (2452022252).

## Conflict of interest

The authors declare that the research was conducted in the absence of any commercial or financial relationships that could be construed as a potential conflict of interest.

## Publisher’s note

All claims expressed in this article are solely those of the authors and do not necessarily represent those of their affiliated organizations, or those of the publisher, the editors and the reviewers. Any product that may be evaluated in this article, or claim that may be made by its manufacturer, is not guaranteed or endorsed by the publisher.

## References

[ref1] Akpa G. Alphonsus C. Dalha S. Yakubu H. Garba Y. (2011). Relationship between litter size and parity of doe in smallholder goat herds in Kano and its environs, Nigeria. Afr. J. Agric. Res. 6, 6212–6216. doi: 10.5897/AJAR11.365

[ref2] Andrade B. G. Bressani F. A. Cuadrat R. R. Cardoso T. F. Malheiros J. M. de Oliveira P. S.. (2022). Stool and ruminal microbiome components associated with methane emission and feed efficiency in nelore beef cattle. Front. Genet. 13:812828. doi: 10.3389/fgene.2022.812828, PMID: 35656319 PMC9152269

[ref3] Appuhamy J. R. N. Knoebel N. A. Nayananjalie W. D. Escobar J. Hanigan M. D. (2012). Isoleucine and leucine independently regulate mTOR signaling and protein synthesis in MAC-T cells and bovine mammary tissue slices. J. Nutr. 142, 484–491. doi: 10.3945/jn.111.152595, PMID: 22298573

[ref4] Asma Z. Sylvie C. Laurent C. Jérôme M. Christophe K. Olivier B.. (2013). Microbial ecology of the rumen evaluated by 454 GS FLX pyrosequencing is affected by starch and oil supplementation of diets. FEMS Microbiol. Ecol. 83, 504–514. doi: 10.1111/1574-6941.12011, PMID: 22974422

[ref5] Beauchemin K. A. Kreuzer M. O’Mara F. McAllister T. A. (2008). Nutritional management for enteric methane abatement: a review. Aust. J. Exp. Agric. 48, 21–27. doi: 10.1071/EA07199

[ref6] Bi Y. Tu Y. Zhang N. Wang S. Zhang F. Suen G.. (2021). Multiomics analysis reveals the presence of a microbiome in the gut of fetal lambs. Gut 70, 853–864. doi: 10.1136/gutjnl-2020-320951, PMID: 33589511 PMC8040156

[ref7] Bi Y. Zeng S. Zhang R. Diao Q. Tu Y. (2018). Effects of dietary energy levels on rumen bacterial community composition in Holstein heifers under the same forage to concentrate ratio condition. BMC Microbiol. 18:69. doi: 10.1186/s12866-018-1213-9, PMID: 29996759 PMC6042446

[ref8] Callahan B. J. McMurdie P. J. Rosen M. J. Han A. W. Johnson A. J. A. Holmes S. P. (2016). DADA2: high-resolution sample inference from Illumina amplicon data. Nat. Methods 13, 581–583. doi: 10.1038/nmeth.3869, PMID: 27214047 PMC4927377

[ref9] Caporaso J. G. Lauber C. L. Walters W. A. Berg-Lyons D. Huntley J. Fierer N.. (2012). Ultra-high-throughput microbial community analysis on the Illumina HiSeq and MiSeq platforms. ISME J. 6, 1621–1624. doi: 10.1038/ismej.2012.8, PMID: 22402401 PMC3400413

[ref10] Carberry C. A. Kenny D. A. Han S. McCabe M. S. Waters S. M. (2012). Effect of phenotypic residual feed intake and dietary forage content on the rumen microbial community of beef cattle. Appl. Environ. Microbiol. 78, 4949–4958. doi: 10.1128/AEM.07759-11, PMID: 22562991 PMC3416373

[ref11] Chen J. Lei X. J. Wang L. Zhang Y. L. Wang D. D. Zhao L. C.. (2022). Effects of rumen-protected leucine on production performance and starch digestion in the small intestine of lactating goats. Anim. Feed Sci. Technol. 287:115270. doi: 10.1016/j.anifeedsci.2022.115270

[ref12] Chen S. Zhou Y. Chen Y. Gu J. (2018). fastp: an ultra-fast all-in-one FASTQ preprocessor. Bioinformatics 34, i884–i890. doi: 10.1093/bioinformatics/bty560, PMID: 30423086 PMC6129281

[ref13] Chilliard Y. Glasser F. Ferlay A. Bernard L. Rouel J. Doreau M. (2007). Diet, rumen biohydrogenation and nutritional quality of cow and goat milk fat. Eur. J. Lipid Sci. Technol. 109, 828–855. doi: 10.1002/ejlt.200700080

[ref14] Christley R. Morgan K. Parkin T. French N. (2003). Factors related to the risk of neonatal mortality, birth-weight and serum immunoglobulin concentration in lambs in the UK. Prev. Vet. Med. 57, 209–226. doi: 10.1016/s0167-5877(02)00235-0, PMID: 12609466

[ref15] De Filippis F. Pasolli E. Tett A. Tarallo S. Naccarati A. De Angelis M.. (2019). Distinct genetic and functional traits of human intestinal *Prevotella copri* strains are associated with different habitual diets. Cell Host Microbe 25, 444.e443–453.e443. doi: 10.1016/j.chom.2019.01.004, PMID: 30799264

[ref16] de Lima L. G. de Souza N. O. B. Rios R. R. de Melo B. A. dos Santos L. T. A. Silva K. D. M.. (2020). Advances in molecular genetic techniques applied to selection for litter size in goats (*Capra hircus*): a review. J. Appl. Anim. Res. 48, 38–44. doi: 10.1080/09712119.2020.1717497

[ref17] Fievez V. Colman E. Castro-Montoya J. Stefanov I. Vlaeminck B. (2012). Milk odd-and branched-chain fatty acids as biomarkers of rumen function—an update. Anim. Feed Sci. Technol. 172, 51–65. doi: 10.1016/j.anifeedsci.2011.12.008

[ref18] Gootwine E. Spencer T. Bazer F. (2007). Litter-size-dependent intrauterine growth restriction in sheep. Animal 1, 547–564. doi: 10.1017/S1751731107691897, PMID: 22444412

[ref19] Graf J. (2014). “The family Rikenellaceae” in The Prokaryotes. eds. Rosenberg E. DeLong E. Lory S. Stackebrandt E. Thompson F. (Berlin: Springer)

[ref20] Haenlein G. (2004). Goat milk in human nutrition. Small Rumin. Res. 51, 155–163. doi: 10.1016/j.smallrumres.2003.08.010

[ref21] Hummel G. Woodruff K. Austin K. Knuth R. Lake S. Cunningham-Hollinger H. (2021). Late gestation maternal feed restriction decreases microbial diversity of the placenta while mineral supplementation improves richness of the fetal gut microbiome in cattle. Animals 11:2219. doi: 10.3390/ani11082219, PMID: 34438676 PMC8388467

[ref22] Jami E. Israel A. Kotser A. Mizrahi I. (2013). Exploring the bovine rumen bacterial community from birth to adulthood. ISME J. 7, 1069–1079. doi: 10.1038/ismej.2013.2, PMID: 23426008 PMC3660679

[ref23] Jami E. White B. A. Mizrahi I. (2014). Potential role of the bovine rumen microbiome in modulating milk composition and feed efficiency. PLoS One 9:e85423. doi: 10.1371/journal.pone.0085423, PMID: 24465556 PMC3899005

[ref24] Jiang X. Lu N. Xue Y. Liu S. Lei H. Tu W.. (2019). Crude fiber modulates the fecal microbiome and steroid hormones in pregnant Meishan sows. Gen. Comp. Endocrinol. 277, 141–147. doi: 10.1016/j.ygcen.2019.04.006, PMID: 30951727

[ref25] Kabel M. A. Yeoman C. J. Han Y. Dodd D. Abbas C. A. de Bont J. A.. (2011). Biochemical characterization and relative expression levels of multiple carbohydrate esterases of the xylanolytic rumen bacterium *Prevotella ruminicola* 23 grown on an ester-enriched substrate. Appl. Environ. Microbiol. 77, 5671–5681. doi: 10.1128/AEM.05321-11, PMID: 21742923 PMC3165261

[ref26] Kamke J. Kittelmann S. Soni P. Li Y. Tavendale M. Ganesh S.. (2016). Rumen metagenome and metatranscriptome analyses of low methane yield sheep reveals a *Sharpea*-enriched microbiome characterised by lactic acid formation and utilisation. Microbiome 4, 56–16. doi: 10.1186/s40168-016-0201-2, PMID: 27760570 PMC5069950

[ref27] Kim J. N. Méndez-García C. Geier R. R. Iakiviak M. Chang J. Cann I.. (2017). Metabolic networks for nitrogen utilization in *Prevotella ruminicola* 23. Sci. Rep. 7:7851. doi: 10.1038/s41598-017-08463-3, PMID: 28798330 PMC5552732

[ref28] Lai F.-N. Zhai H.-L. Cheng M. Ma J.-Y. Cheng S.-F. Ge W.. (2016). Whole-genome scanning for the litter size trait associated genes and SNPs under selection in dairy goat (*Capra hircus*). Sci. Rep. 6, 1–12. doi: 10.1038/srep38096, PMID: 27905513 PMC5131482

[ref29] Li F. Li C. Chen Y. Liu J. Zhang C. Irving B.. (2019). Host genetics influence the rumen microbiota and heritable rumen microbial features associate with feed efficiency in cattle. Microbiome 7:92. doi: 10.1186/s40168-019-0699-1, PMID: 31196178 PMC6567441

[ref30] Li F. Li Z. Li S. D. Ferguson J. Cao Y. Yao J.. (2014). Effect of dietary physically effective fiber on ruminal fermentation and the fatty acid profile of milk in dairy goats. J. Dairy Sci. 97, 2281–2290. doi: 10.3168/jds.2013-6895, PMID: 24508430

[ref31] Liu Y. Wu H. Liu C. Zhou Z. (2022). Rumen microbiome and metabolome of high and low residual feed intake angus heifers. Front. Vet. Sci. 9:812861. doi: 10.3389/fvets.2022.812861, PMID: 35400092 PMC8993041

[ref32] Ma C. Zhang W. Gao Q. Zhu Q. Song M. Ding H.. (2020). Dietary synbiotic alters plasma biochemical parameters and fecal microbiota and metabolites in sows. J. Funct. Foods 75:104221. doi: 10.1016/j.jff.2020.104221

[ref33] Ma H. Zhang W. Zhu X. Song W. Liu J. Jia Z. (2010). Effects of rumen-protected tryptophan on growth performance, fibre characteristics, nutrient utilization and plasma essential amino acids in cashmere goats during the cashmere slow growth period. Livest. Sci. 131, 227–233. doi: 10.1016/j.livsci.2010.04.005

[ref34] MacHugh D. E. Bradley D. G. (2001). Livestock genetic origins: goats buck the trend. Proc. Natl. Acad. Sci. U. S. A. 98, 5382–5384. doi: 10.1073/pnas.111163198, PMID: 11344280 PMC33220

[ref35] Maeng W. Van Nevel C. Baldwin R. Morris J. (1976). Rumen microbial growth rates and yields: effect of amino acids and protein. J. Dairy Sci. 59, 68–79. doi: 10.3168/jds.S0022-0302(76)84157-4, PMID: 1249281

[ref36] Magoč T. Salzberg S. L. (2011). FLASH: fast length adjustment of short reads to improve genome assemblies. Bioinformatics 27, 2957–2963. doi: 10.1093/bioinformatics/btr507, PMID: 21903629 PMC3198573

[ref37] Matthews C. Crispie F. Lewis E. Reid M. O’Toole P. W. Cotter P. D. (2019). The rumen microbiome: a crucial consideration when optimising milk and meat production and nitrogen utilisation efficiency. Gut Microbes 10, 115–132. doi: 10.1080/19490976.2018.1505176, PMID: 30207838 PMC6546327

[ref38] Mccann J. C. Wiley L. M. David F. T. Rouquette F. M. Tedeschi L. O. Dan Z. (2014). Relationship between the rumen microbiome and residual feed intake-efficiency of Brahman bulls stocked on bermudagrass pastures. PLoS One 9:e91864. doi: 10.1371/journal.pone.0091864, PMID: 24642871 PMC3958397

[ref39] Myer P. R. Smith T. P. Wells J. E. Kuehn L. A. Freetly H. C. (2015). Rumen microbiome from steers differing in feed efficiency. PLoS One 10:e0129174. doi: 10.1371/journal.pone.0129174, PMID: 26030887 PMC4451142

[ref40] O’Hara E. Neves A. L. Song Y. Guan L. L. (2020). The role of the gut microbiome in cattle production and health: driver or passenger? Annu. Rev. Anim. Biosci. 8, 199–220. doi: 10.1146/annurev-animal-021419-083952 32069435

[ref41] Onodera R. (2003). Essentiality of histidine in ruminant and other animals including human beings. Asian Australas. J. Anim. Sci. 16, 445–454. doi: 10.5713/ajas.2003.445

[ref42] Paz H. A. Hales K. E. Wells J. E. Kuehn L. A. Freetly H. C. Berry E. D.. (2018). Rumen bacterial community structure impacts feed efficiency in beef cattle. J. Anim. Sci. 96, 1045–1058. doi: 10.1093/jas/skx081, PMID: 29617864 PMC6093515

[ref43] Pulina G. Milán M. Lavín M. Theodoridis A. Morin E. Capote J.. (2018). Invited review: current production trends, farm structures, and economics of the dairy sheep and goat sectors. J. Dairy Sci. 101, 6715–6729. doi: 10.3168/jds.2017-14015, PMID: 29859690

[ref44] Rowe M. Veerus L. Trosvik P. Buckling A. Pizzari T. (2020). The reproductive microbiome: an emerging driver of sexual selection, sexual conflict, mating systems, and reproductive isolation. Trends Ecol. Evol. 35, 220–234. doi: 10.1016/j.tree.2019.11.004, PMID: 31952837

[ref45] Shabat S. K. B. Sasson G. Doron-Faigenboim A. Durman T. Yaacoby S. Berg Miller M. E.. (2016). Specific microbiome-dependent mechanisms underlie the energy harvest efficiency of ruminants. ISME J. 10, 2958–2972. doi: 10.1038/ismej.2016.62, PMID: 27152936 PMC5148187

[ref46] Sun X. Gibbs S. (2012). Diurnal variation in fatty acid profiles in rumen digesta from dairy cows grazing high-quality pasture. Anim. Feed Sci. Technol. 177, 152–160. doi: 10.1016/j.anifeedsci.2012.08.013

[ref47] Tong F. Wang T. Gao N. L. Liu Z. Cui K. Duan Y.. (2022). The microbiome of the buffalo digestive tract. Nat. Commun. 13:823. doi: 10.1038/s41467-022-28402-9, PMID: 35145088 PMC8831627

[ref48] Tong J.-J. Zhang H. Jia W. Yun L. Mao S.-Y. Xiong B.-H.. (2020). Effects of different molecular weights of chitosan on methane production and bacterial community structure *in vitro*. J. Integr. Agric. 19, 1644–1655. doi: 10.1016/S2095-3119(20)63174-4

[ref49] Van Houtert M. (1993). The production and metabolism of volatile fatty acids by ruminants fed roughages: a review. Anim. Feed Sci. Technol. 43, 189–225. doi: 10.1016/0377-8401(93)90078-X

[ref50] Vlaeminck B. Fievez V. Cabrita A. Fonseca A. Dewhurst R. (2006). Factors affecting odd-and branched-chain fatty acids in milk: a review. Anim. Feed Sci. Technol. 131, 389–417. doi: 10.1016/j.anifeedsci.2006.06.017

[ref51] Wallace R. J. Sasson G. Garnsworthy P. C. Tapio I. Gregson E. Bani P.. (2019). A heritable subset of the core rumen microbiome dictates dairy cow productivity and emissions. Sci. Adv. 5:eaav8391. doi: 10.1126/sciadv.aav8391, PMID: 31281883 PMC6609165

[ref52] Wang S. Yao B. Gao H. Zang J. Tao S. Zhang S.. (2019). Combined supplementation of *lactobacillus fermentum* and *Pediococcus acidilactici* promoted growth performance, alleviated inflammation, and modulated intestinal microbiota in weaned pigs. BMC Vet. Res. 15:239. doi: 10.1186/s12917-019-1991-9, PMID: 31291967 PMC6617942

[ref53] Wu D. Xu L. Tang S. Guan L. He Z. Guan Y.. (2016). Influence of oleic acid on rumen fermentation and fatty acid formation *in vitro*. PLoS One 11:e0156835. doi: 10.1371/journal.pone.0156835, PMID: 27299526 PMC4907511

[ref54] Xue M. Sun H. Wu X. Guan L. L. Liu J. (2018). Assessment of rumen microbiota from a large dairy cattle cohort reveals the pan and core bacteriomes contributing to varied phenotypes. Appl. Environ. Microbiol. 84:e00970-00918. doi: 10.1128/AEM.00970-18, PMID: 30054362 PMC6146982

[ref55] Xue M. Sun H. Wu X. Guan L. Liu J. (2019). Assessment of rumen bacteria in dairy cows with varied milk protein yield. J. Dairy Sci. 102, 5031–5041. doi: 10.3168/jds.2018-15974, PMID: 30981485

[ref56] Xue M.-Y. Sun H.-Z. Wu X.-H. Liu J.-X. Guan L. L. (2020). Multi-omics reveals that the rumen microbiome and its metabolome together with the host metabolome contribute to individualized dairy cow performance. Microbiome 8, 64–19. doi: 10.1186/s40168-020-00819-8, PMID: 32398126 PMC7218573

[ref57] Xue B. Wu M. Yue S. Hu A. Li X. Hong Q.. (2022). Changes in rumen bacterial community induced by the dietary physically effective neutral detergent fiber levels in goat diets. Front. Microbiol. 13:820509. doi: 10.3389/fmicb.2022.820509, PMID: 35479630 PMC9035740

[ref58] Xue M.-Y. Xie Y.-Y. Zhong Y. Ma X.-J. Sun H.-Z. Liu J.-X. (2022). Integrated meta-omics reveals new ruminal microbial features associated with feed efficiency in dairy cattle. Microbiome 10, 32–14. doi: 10.1186/s40168-022-01228-9, PMID: 35172905 PMC8849036

[ref59] Zhang C. Yang L. Shen Z. (2008). Variance components and genetic parameters for weight and size at birth in the boer goat. Livest. Sci. 115, 73–79. doi: 10.1016/j.livsci.2007.06.008

[ref60] Zhang Y. Zhang X. Li F. Li C. Li G. Zhang D.. (2021). Characterization of the rumen microbiota and its relationship with residual feed intake in sheep. Animal 15:100161. doi: 10.1016/j.animal.2020.100161, PMID: 33785185

