# Supplementary material for: Litter size influences rumen microbiota and fermentation efficiency, thus determining host early growth in goats
Source: Front Microbiol. 2023 Jan 20;14:1098813. doi: 10.3389/fmicb.2023.1098813 (PMC9895106; doi:10.3389/fmicb.2023.1098813)
Supplement: Supplementary file 1 [file Data_Sheet_1.docx]

**Supplementary table**

Table S1. Feed ingredient and chemical composition of the young goat

| Ingredients, % of DM | Content | Chemical composition | Content |
| --- | --- | --- | --- |
| Alfalfa hay | 39.9 | DM, % | 57.40 |
| Corn silage | 20.6 | CP, % of DM | 15.56 |
| Corn | 20.9 | Starch, % of DM | 21.68 |
| Wheat bran | 10.7 | NDF, % of DM | 30.35 |
| Soybean meal | 5.9 | ADF, % of DM | 15.61 |
| NaHCO_3_ | 0.3 |  |  |
| CaHPO_4_ | 0.3 |  |  |
| NaCl | 1.3 |  |  |
| Premix^1^ | 0.1 |  |  |

^1^Premix contained (per kg) the following: Cu, 2,925 mg; Fe, 3,900 mg; Zn, 2,750 mg; Mn, 800 mg; vitamin A, 1,500 kIU; vitamin D3, 500 kIU; and vitamin E, 5,500 IU.

**Supplementary figure**

Fig. S1 Effect of litter size on birth weight of newborns (A), and the difference in DMI of young goats between different groups (B). Data are presented as mean ± SEM.

Fig. S2 Compositions of the rumen microbiota among the different groups at phylum level. (A) Relative abundance of major phyla, (B) Firmicutes, (C) Bacteroidota, (D) the Firmicutes: Bacteroidota ratio. Data are presented as mean ± SEM, **P* < 0.05, ***P* < 0.01.
